# Cell-Wall-Degrading Enzymes Required for Virulence in the Host Selective Toxin-Producing Necrotroph *Alternaria alternata* of Citrus

**DOI:** 10.3389/fmicb.2019.02514

**Published:** 2019-11-22

**Authors:** Haijie Ma, Bin Zhang, Yunpeng Gai, Xuepeng Sun, Kuang-Ren Chung, Hongye Li

**Affiliations:** ^1^Key Lab of Molecular Biology of Crop Pathogens and Insects, Ministry of Agriculture, Institute of Biotechnology, Zhejiang University, Hangzhou, China; ^2^Department of Plant Pathology, College of Agriculture and Natural Resources, National Chung-Hsing University, Taichung, Taiwan

**Keywords:** citrus, cutinase, toxin, virulence, *Ste12*, CAZymes, pathogenicity

## Abstract

The necrotrophic fungal pathogen *Alternaria alternata* attacks many citrus species, causing brown spot disease. Its pathogenic capability depends primarily on the production of host-selective ACT toxin. In the current study a Ste12 transcription factor was characterized to be required for conidial formation and the production of cell-wall-degrading enzymes (CWDEs) in the tangerine pathotype of *A. alternata*. The Ste12 deficiency strain (ΔSte12) retained wild-type growth, ACT toxin production, and sensitivity to oxidative and osmotic stress. However, pathogenicity tests assayed on detached Dancy leaves revealed a marked reduction in virulence of ΔSte12. Transcriptome and quantitative RT-PCR analyses revealed that many genes associated with Carbohydrate-Active Enzymes (CAZymes) were downregulated in ΔSte12. Two cutinase-coding genes (*AaCut3* and *AaCut7*) regulated by Ste12 were individually and simultaneously inactivated. The *AaCut3* or *AaCut7* deficiency strain unchanged in cutinase activities and incited wild-type lesions on Dancy leaves. However, the strain carrying an *AaCut3 AaCut7* double mutation produced and secreted significantly fewer cutinases and incited smaller necrotic lesions than wild type. Not only is the host-selective toxin (HST) produced by *A. alternata* required for fungal penetration and lesion formation, but so too are CWDEs required for full virulence. Overall, this study expands our understanding of how *A. alternata* overcomes citrus physical barriers to carry out successful penetration and colonization.

## Introduction

Plant pathogenic fungi utilize a wide range of arsenals to penetrate and colonize their hosts. Toxins and cell-wall-degrading enzymes (CWDEs) are the two most common weapons utilized by fungal necrotrophs to attack host plants. The tangerine pathotype of *Alternaria alternata* produces a host-selective toxin (HST) called ACT (Ito et al., 2004), which primarily affects tangerines (*Citrus reticulata* Blanco), grapefruit (*C. paradisi* Macfad.), and hybrids of grapefruit and tangerine, and tangerine and sweet orange [*C. sinensis* (L.) Osbeck] (Timmer and Peever, 1997). ACT containing a 9,10-epoxy-8-hydroxy-9-methyl-decatrienoic acid structure induces rapid electrolyte leakage from citrus cells (Kohmoto et al., 1993). Although the exact targets of ACT remain unknown, ACT is a pathogenicity determinant that is required for the formation of necrotic lesions (Miyamoto et al., 2008).

In addition to ACT toxin, previous studies have demonstrated that the ability to detoxify toxic reactive oxygen species (ROS) is critical to colonize host tissue. Several factors or pathways required for ROS detoxification have also been identified in *A. alternata*. Those include the glutaredoxin/thioredoxin systems, the Yap1 transcription factor, the Skn7 response regulator, the NADPH oxidase-mediated H_2_O_2_ production, the siderophore-mediated iron homeostasis, methionine biosynthesis regulator, and the Hog1 mitogen-activated protein kinase (MAPK)-mediated pathway (Yang and Chung, 2013; Chung, 2014; Yang et al., 2016; Ma et al., 2018; Gai et al., 2019).

Mitogen-activated protein kinase-mediated phosphorelay pathways, which function in sensing environmental stimuli and gene regulation are important signaling pathways in eukaryotes (Pelech and Sanghera, 1992). Unlike Hog1, the Fus3 MAPK-mediated signaling pathway of *A. alternata* is not required for oxidative stress resistance. The *A. alternata* Fus3 has been demonstrated to be required for vegetative growth, conidiation, pathogenicity, osmotic adjustment, resistance to copper fungicides, the production of hydrolytic enzymes and melanin, and virulence (Lin and Chung, 2010). In the budding yeast *Saccharomyces cerevisiae*, Ste12 is directly regulated by the Fus3/Kss1 MAPK cascade pathway and plays an important role in mating and invasive growth (Madhani et al., 2001). In filamentous fungi, Ste12 plays various roles in different species, including fertility, development, conidiation, and/or virulence (Hoi and Dumas, 2010; Rispail and Di Pietro, 2010). However, the role of Ste12 in the tangerine pathotype of *A. alternata* remains unknown.

Studies in the wheat pathogen *Fusarium graminearum* have revealed that genes encoding CWDEs are regulated by Ste12 (Gu et al., 2015). Many fungi including *Alternaria* species are known to produce a wide array of CWDEs (ten Have et al., 2002; Kubicek et al., 2014). CWDEs are required for virulence in many phytopathogenic fungi including *Magnaporthe grisea*, *Curvularia lunata*, *Valsa mali*, and *F. graminearum* (Skamnioti and Gurr, 2007; Van Vu et al., 2012; Liu et al., 2016; Wu et al., 2017). However, the pathological role of CWDEs in the toxin-producing *Alternaria* species remains elusive as the toxin is the most prominent determinant of virulence in *Alternaria* pathogenesis.

Because the tangerine pathotype of *A. alternata* heavily depends on the production of HST to attack its host, CWDEs have been suggested but never conclusively demonstrated to be required for *A. alternata* pathogenesis (Tanabe et al., 1988; Isshiki et al., 2001). As part of the efforts to better understand the mechanisms underlying *Alternaria* pathogenesis, we characterize a Ste12 transcription factor downstream of Fus3. The results indicate that Ste12 is required for conidiation and virulence in the tangerine pathotype of *A. alternata*. Mutational inactivation of *Ste12* resulted in fungal strains that reduced virulence and had relatively lower activities of cutinase, cellulase, and pectate lyase. Genetic evidence was also provided to define the critical role of cutinases in *A. alternata* pathogenesis. This study widens our understanding of how *A. alternata* conquers citrus physical barriers to achieve successful penetration and establishment, which could potentially contribute to long-term sustainable management of the disease.

## Materials and Methods

### Fungal Strains and Growth Conditions

The wild-type Z7 strain of *A. alternata* (Fr.) Keissler used in the mutagenesis experiments was isolated from a diseased Ougan (*Citrus suavissima* Hort. ex Tanaka) leaf in Zhejiang, China and has been previously characterized (Wang et al., 2016). ΔΔACTT6 strain, which produced no detectable ACT toxin and markedly reduced in necrotic lesions on Dancy leaves, was previously created by deleting two copies of *ACTT6* required for ACT toxin biosynthesis in the *A. alternata* (*unpublished*). Fungal strains were cultured on potato dextrose agar (PDA) at 28°C. Conidia were harvested from fungal cultures grown on V8 juice agar or PDA for 7 days by immersing, scraping with sterile water, and passing through two layers of sterile cheesecloth. Mycelium was collected from 36-h liquid cultures by passing through cheesecloth and used for purification of DNA or RNA. Regeneration medium (RMM) used to recover transformants was prepared as described (Chung et al., 2002). Assays for sensitivity to chemicals were performed on PDA amended with test compounds. Fungal strains were grown in Richard’s solution (Kohmoto et al., 1993) for 14–28 days for the production of a host-specific ACT toxin.

### Targeted Gene Disruption and Genetic Complementation

The *A. alternata Ste12* gene (*AaSte12*) was mutated by integrating a bacterial phosphotransferase B gene (*HYG*) cassette under control of the *Aspergillus nidulans TrpC* gene promoter and terminator in the genome of Z7 using a split marker approach as described (Supplementary Figure S2). Two fragments *5′-Ste12:HY/g* and *h/YG:Ste12-3′* overlapping within the *HYG* gene were generated by two-round PCR, combined, and transformed into protoplasts prepared from Z7 using CaCl_2_ and polyethylene glycol as previously described (Chung et al., 2002; Chen et al., 2012). Fungal transformants were recovered from RMM containing 100 μg/ml hygromycin and examined for gene deletion by PCR with an *AaSte12* primer that was not localized in the split marker fragments pairing with a *Hyg*-specific primer. An *AaSte12* DNA fragment including its endogenous promoter was amplified by PCR from the Z7 genome and translationally fused with a GFP-coding gene, which fused further with a *Neo* gene conferring resistance to neomycin to yield an *AaSte12*:*GFP*:neo fragment. *AaSte12*:*GFP*:neo was transformed into protoplasts prepared from an *AaSte12*-deletion mutant for genetic complementation. Oligonucleotide primers used in this study are listed in Supplementary Table S8.

Similar strategies using split *Neo* marker gene fragments were used to disrupted *AaCut3* (AALT_g1915) in the Z7 genome (Supplementary Figure S5). *AaCut7* (AALT_g8018) was disrupted using a split *Hyg* marker gene fragments (Supplementary Figure S6). ΔΔCut3/7 deleted at both *AaCut3* and *AaCut7* was generated by transforming two overlapping *AaCut7:Neo* into protoplasts prepared from one of ΔCut3. Fungal transformants defective at each locus was examined by PCR with a gene-specific primer not present in the split marker fragment pairing with a *HYG* or *Neo* gene primer.

### Transcriptome Analysis

Wild type and ΔAaSte12 each with two replicates were cultured in potato dextrose broth (PDB) for 36 h (120 rpm, 28°C). RNA was extracted using Axygen RNA purification kit (Capital Scientific, Union City, CA, United States). The libraries were constructed using an IlluminaTruSeq RNA Sample Preparation Kit. Sequencing was performed using an Illumina HIseq2500 platform, generating 150 bp paired-end reads. Adaptors and low-quality reads were removed using Trimmomatic Ver 0.36 (Bolger et al., 2014). Sequences were mapped to the reference genome using TopHat2 (Kim et al., 2013) and the number of reads mapped to each gene was determined by HTSeq (Anders et al., 2015). Differential expression analysis was determined using DESEQ2 (Anders and Huber, 2012) based on the overall transcript counts after normalization. Differentially expressed genes (DEGs) were defined by the False Discovery rate (FDR) less than 0.01 and the absolute value of log2 fold change (log2FC) greater than 1. Differentially expressed genes were annotated by searching against the NCBI nr databases. Gene ontology (GO) and Kyoto Encyclopedia of Genes and Genomes (KEGG) pathway were performed using clusterProfiler v3.6 (Yu et al., 2012). All genes with annotation of KEGG and NR are listed in Supplementary Table S9. Gene clusters associated with secondary metabolites were predicted using anti-SMASH 4.0 (Blin et al., 2017). Protein domains were predicted in SMART database available at http://smart.embl-heidelberg.de/. Maximum likelihood phylogenies were constructed using MEGA 6.0 and iTOL available at https://itol.embl.de/. Carbohydrate-active enzymes (CAZymes) were predicted using dbCAN meta server^1^. The transcriptomes raw data (Z7-1: SRR8556263; Z7-2: SRR8556264; ΔSte12-1: SRR8556261; ΔSte12-2: SRR8556262) have been deposited in NCBI’s Sequence Read Archive.

### Gene Expression Analyses

Relative expression of the genes in *A. alternata* strains cultured in PDB amended with or without citrus leaves was quantified by quantitative RT-PCR using a 7300 Real Time PCR system (Applied Biosystems, Carlsbad, CA, United States). Fungal RNA was extracted with an Axygen RNA purification kit (Capital Scientific). First strand DNA was synthesized from RNA using a Prime Script RT reagent kit (Takara, Shiga, Japan) and used for PCR amplification. The relative expression levels to the wild type after normalization to the actin-coding gene (accession number: KP341672) were calculated from three independent reactions using a comparative C_T_ method as previously described (Sun et al., 2011).

### Microscopy

Fungal conidia and hyphae were examined with a Nikon microscope equipped with a LV100ND image system (Nikon, Japan). Nuclei in fungal hyphae grown in liquid medium for 24 h were stained with 1 μg/ml 4′,6-diamidino-2-phenylindole (DAPI) (Sigma, St. Louis, MO, United States) for 3∼5 min at room temperature and then washed with ddH_2_O_2_. GFP fluorescence was examined with a Zeiss LSM780 confocal microscope (Gottingen, Niedersachsen, Germany).

### Assays for Cell-Wall-Degrading Enzymes (CWDEs)

To determine the activities of CWDEs, fungal strains were cultured in induction medium amended with 0.25% nitrobenzoic acid (for cutinase activities), 1% pectin (for pectate lyases), or 1.76% sodium carboxymethyl cellulose (for cellulases) at 22°C for 7 days. Cellulases and pectate lyases were determined by measuring the amounts of reducing sugar released from 1% citrus pectin and 1.76% sodium carboxymethyl cellulose, respectively, reacted with dinitrosalicylic acid (DNS)-containing reagent, and measured spectrophotometer at wavelength OD_540 nm_ as described (Mankarios and Friend, 1980; Marcus et al., 1986; Kapat et al., 1998). The regression line and correlation coefficient (*r*^2^ > 0.999) were established using polygalacturonic acid (PGA) or glucose as a standard. One unit of enzyme activity is defined as that required to liberate 1 μmole of glucose or PGA from substrate per minute. Cutinase activities were determined after forming a yellow color with 5 mM *para*-nitrophenyl butyrate (PNPB) dissolved in 50 mM potassium phosphate (pH 7.0) for 10 min and measuring at A_405_ as described (Dickman and Patil, 1986; Davies et al., 2000). One unit of cutinase was defined by releasing 1 μmole of *p*-nitrophenol per minute.

### Virulence Assays

Fungal virulence was assessed on detached Dancy (*Citrus reticulata*) leaves inoculated by placing 4-mm dia. agar plugs carrying fungal mycelium or 10 μl conidial suspensions (10^4^ spores/ml) on citrus leaves. Virulence was also assayed on Dancy leaves that were wounded with a fine needle prior to inoculation. Each treatment contained at least 10 leaves and experiments were repeated twice. The inoculated leaves were kept in a plastic box at 26°C for 2–4 days for lesion development. For ACT purification, fungal strains were cultured in a Richard’s broth (1 L) at 26°C for 14 to 28 days on a rotary shaker set at 180 rpm. ACT toxin was purified from cultural filtrates after removing mycelium by filtrating through a miracloth. Cultural filtrates were passed through a column pre-packed with Amberlite XAD-2 resins, extracted with ethyl acetate as described (Kohmoto et al., 1993). After drying, ACT was dissolved in methanol and used for bioassays on detached Dancy leaves by placing 10 μl crude extracts on each spot that was pre-wounded with a fine needle. Leaves treated with methanol were used as mocked controls.

### HPLC Analysis

For HPLC analysis, pH of cultural filtrates was adjusted to 5.5 using 10% phosphate buffer (NaH_2_PO_4_ or Na_2_HPO_4_). Cultural filtrates were mixed with XAD-2 resins (1:30, w:v) for 2 h and loaded into a column. ACT was eluted from Amberlite XAD-2 resins with 40 ml methanol. Methanol was dried in a rotary evaporator and ACT was dissolved in 1 ml methanol. ACT toxin was separated through a XbridgeTM18.5 column (4.6 × 250 mm) in a Model 880-PU HPLC system (Japan Spectroscopic, Tokyo, Japan) using a gradient solvent system composed of methanol/0.1% acetic acid at a flow rate set at 1 ml per min. ACT was detected by a UV detector with absorbance set at 290 nm. Extracts purified from Richard’s medium alone (minus fungal strain) in a similar manner were used as a negative control.

### Statistical Analysis

The significance of treatments was determined by analysis of variance and means separated by Ducan’s test (*P* < 0.05).

## Results

### AaSte12 Is Required for Conidiation

**FIGURE 1:**
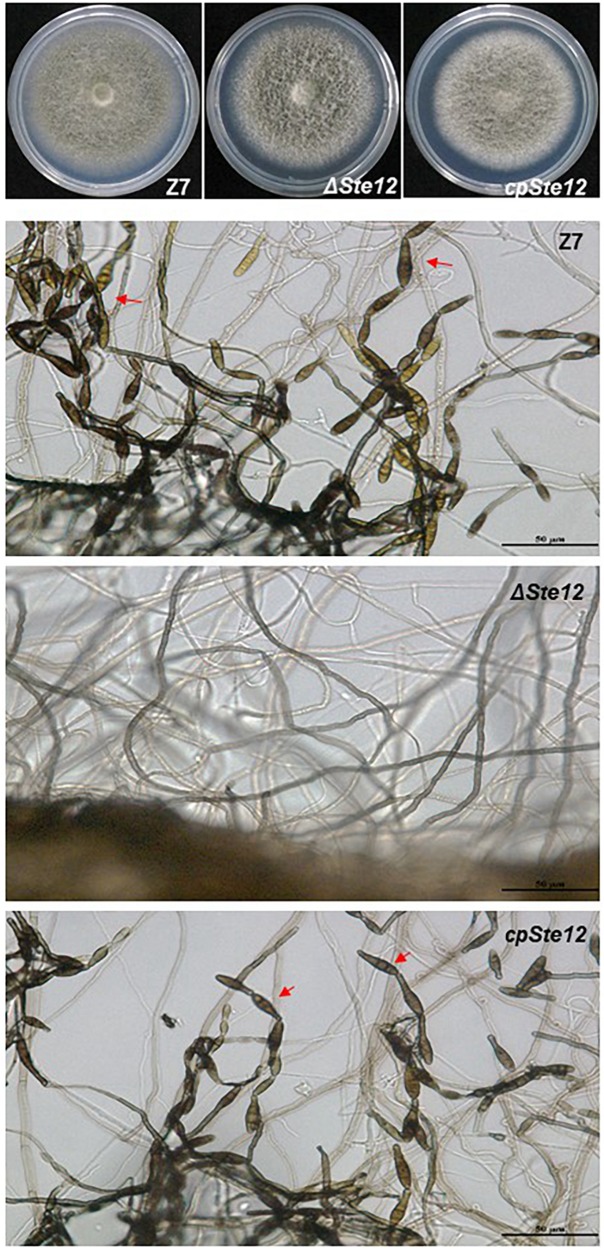
AaSte12 is required for conidiation but dispensable for vegetative growth in *A. alternata*. Fungal strains: Z7 (wild type), ΔSte12, and cpSte12 (complementation strain) were grown on PDA for 4 days. The production of conidia (indicated by red arrows) was examined microscopically. Both Z7 and cpSte12 strains produced dark-pigmented conidia, which were completely absent in ΔSte12.

**FIGURE 2:**
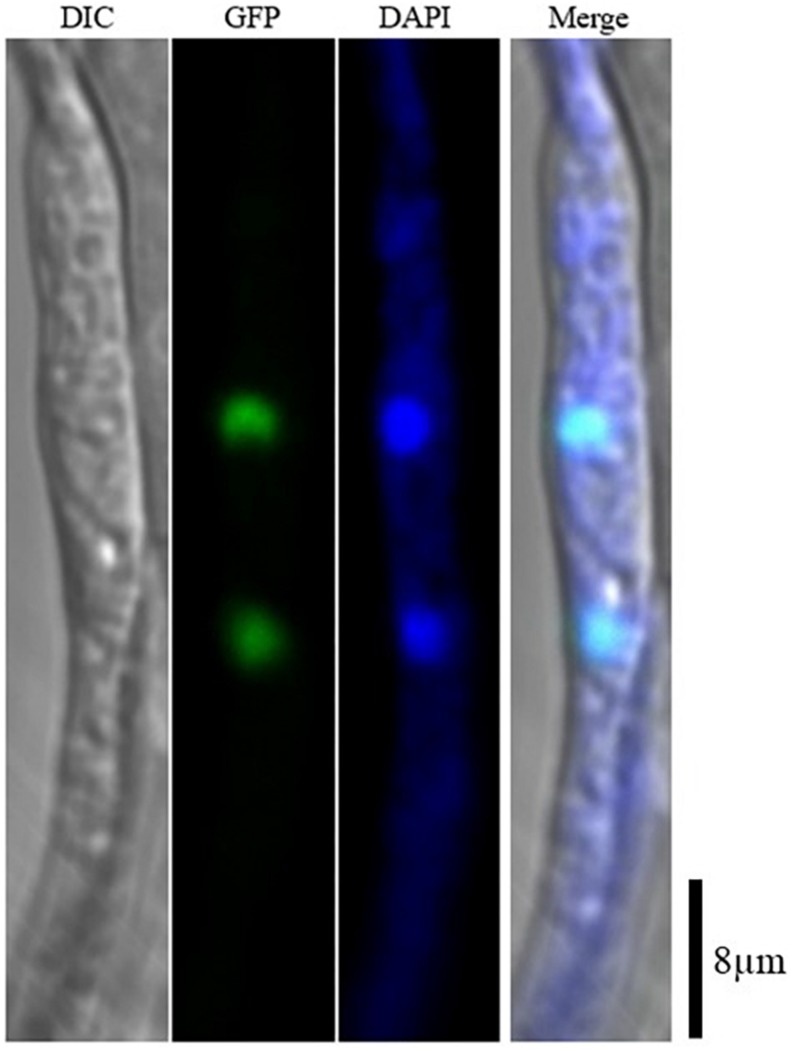
Subcellular localization of AaSte12 in *A. alternata*. A functional copy of *AaSte12*:*GFP*:neo was transformed into protoplasts prepared from a ΔSte12 strain. The resultant strains were examined with a Zeiss LSM780 confocal microscope. Nuclei in fungal hyphae were stained with 4′,6-diamidino-2-phenylindole (DAPI).

*AaSte12* sequence was retrieved from completely sequenced genome of *A. alternata*. *AaSte12* contains a 2061-bp open reading frame (ORF), which encodes a 686 amino acid polypeptide after splicing four small introns (55, 52, 102, and 54 bp). A STE domain and two C_2_H_2_ zinc finger domains, commonly present in Ste12 orthologs, were also found in AaSte12 (Supplementary Figure S1). A split marker approach was performed to delete *AaSte12* in the genome of the Z7 strain, resulting in the identification of two fungal strains designated ΔSte12-B and ΔSte12-D (Supplementary Figure S2). ΔSte12 displayed wild-type growth on artificial medium but completely failed to produce conidia (Figure 1). Transformation of a functional copy of *AaSte12* translationally fused with GFP under control of its endogenous promoter (*AaSte12*:*GFP*:neo) into protoplasts prepared from ΔSte12-B identified a te12 strain that restored conidial formation at levels similar to that of wild type. ΔSte12 displayed wild-type sensitivity to H_2_O_2_, KCl, NaCl, LiCl, and sorbitol (data not shown). Fluorescence microscopy examination of te12 expressing an *AaSte12*:*GFP*:*neo* construct revealed distinct green foci that corresponded to the DAPI-staining nuclei (Figure 2).

### AaSte12 Plays a Role in Fungal Penetration

**FIGURE 3:**
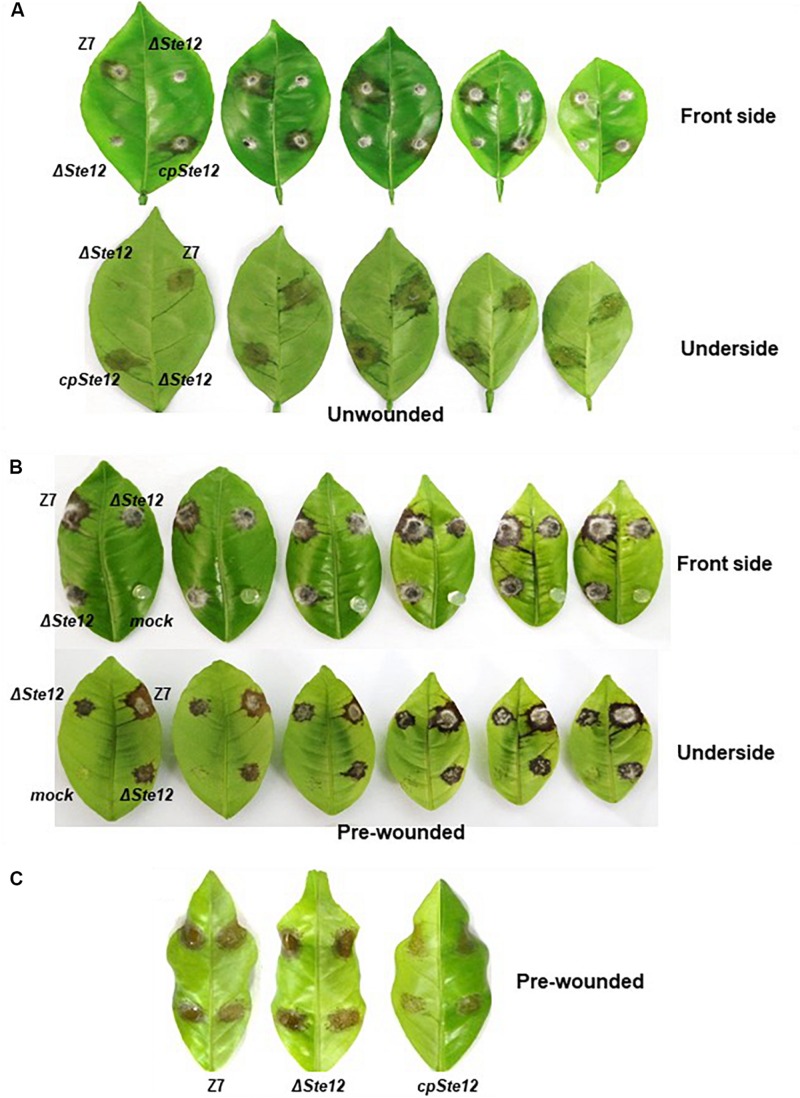
AaSte12 is indispensable for virulence of *A. alternata* to the citrus. Fungal strains: Z7 (wild type), ΔSte12, and cpSte12 (complementation strain) were grown on PDA for 4 days. **(A)** Fungal pathogenicity was assayed on detached Dancy leaves (*n* = 10) by tooth-pick transferring agar plugs carrying fungal propagules to leaf surface (unwounded). Necrotic lesions were recorded at 2 dpi. **(B)** Pathogenicity was also assayed on Dancy leaves that were wounded with a fine needle before inoculation. **(C)** A leaf necrosis assay for the toxicity of ACT toxin by placing 10 μl extracts purified from cultural filtrates of fungal strains on detached Dancy leaves that were wounded before treatment. Leaves treated with agar plugs without fungi or methanol were used as mock controls (not shown). Leaves were kept in a plastic box for lesion development.

Because ΔSte12 was unable to produce conidia, fungal virulence was assessed by placing agar plugs carrying mycelium on detached Dancy leaves. Unlike the wild-type strain, ΔSte12 failed to incite visible lesions on Dancy leaves 2 days post inoculation (dpi). CpSte12 induced necrotic lesions at rates and magnitudes resemble wild type 2 dpi. ΔSte12 was able to induce necrotic lesions on detached Dancy leaves that were pre-wounded before inoculation 2 dpi (Figures 3A,B). The necrotic lesions induced by ΔSte12 were much smaller than those induced by wild type, however.

### AaSte12 Has No Roles in the Production of Host Selective Toxin

**FIGURE 4:**
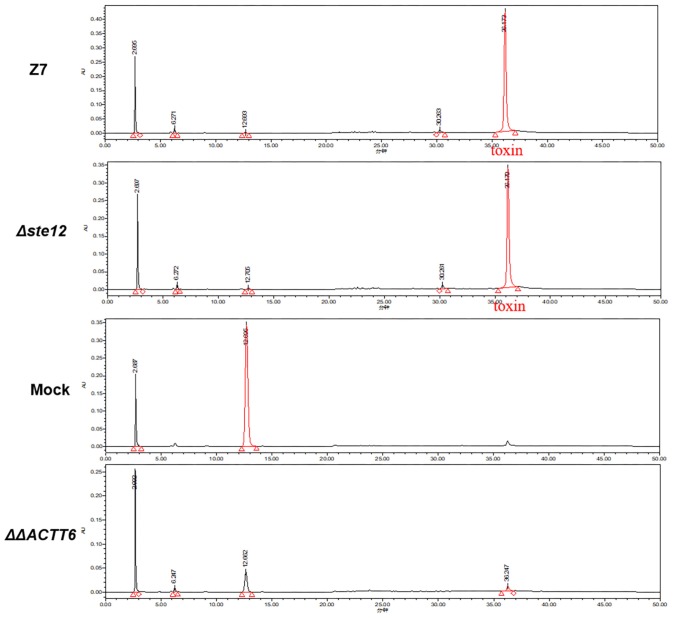
AaSte12 plays no roles in the production of ACT toxin. Fungal strains: Z7 (wild type), ΔSte12, and ΔΔACTT6 (defective for both *ACTT_6-1* and *ACTT_6-2* genes involved in the biosynthesis of ACT) were grown in Richard’s solution for 14 days. ACT was extracted with Amberlite XAD-2 resins, dissolved in methanol, and analyzed by HPLC. Extracts purified from Richard’s medium alone (minus fungal strain) or the ΔΔACTT6 double mutation strain with a similar manner were used as negative controls. A distinct peak with the retention time of 36.5 min was detected in the extracts purified from wild type and ΔSte12. The peak was barely detectable in samples purified from medium alone or ΔΔACTT6.

Bioassays of cultural filtrates prepared from wild type, ΔSte12, and cpSte12 in pre-wounded Dancy leaves all resulted in necrotic lesions with similar sizes (Figure 3C). ACT was purified further from cultural filtrates and analyzed by HPLC, confirming further that ΔSte12 produced ACT at levels closely resemble those produced by wild type (Figure 4). ΔΔACTT6 strain deleted in both *ACTT_6-1* and *ACTT_6-2* genes involved in the biosynthesis of ACT failed to accumulate the toxin.

### Transcriptome Analysis Defines the Global Regulatory Role of AaSte12

**FIGURE 5:**
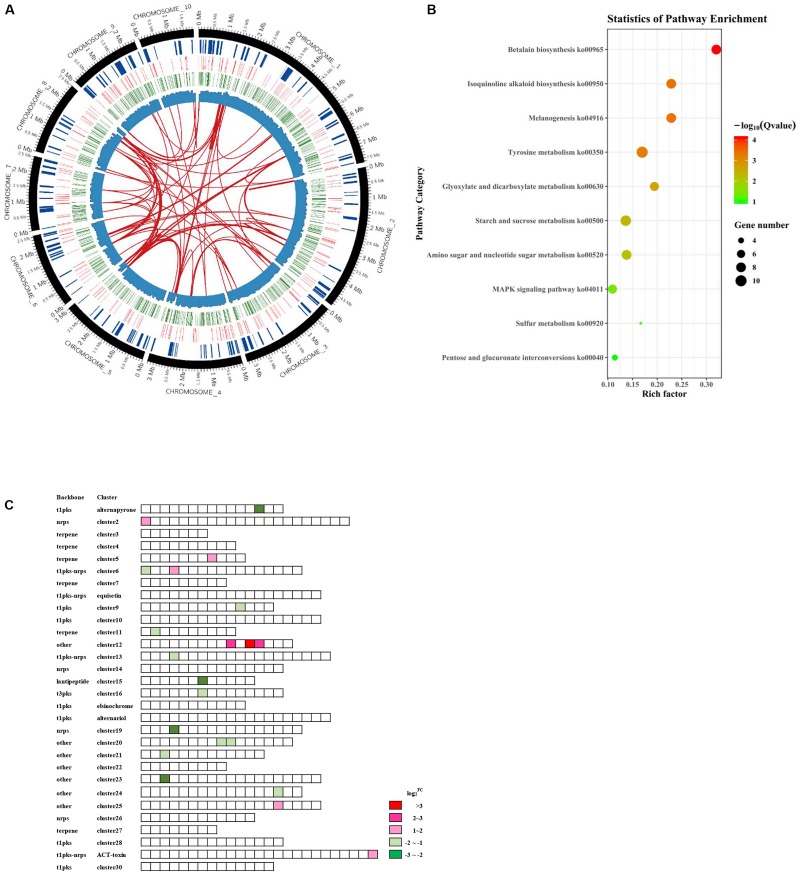
Transcriptome analysis reveals the global regulatory functions of AaSte12 in *A. alternata*. **(A)** Circos plots showing DEGs in the Ste12 deficiency strain (ΔSte12) in relation to those of the wild-type strain (Z7). The circles, from periphery to the core, represent the chromosomes (excluding conditionally dispensable chromosome), the secondary metabolite gene clusters, the DEGs with upregulation in red and downregulation in green, and the GC content, respectively. Gene duplication is shown in the center. **(B)** Scatter plots of KEGG pathway enrichment based on statistic analysis of downregulated genes in ΔSte12. Rich Factor represents the ratio of the number of DEGs annotated in the pathway term in relation to the number of all genes annotated in the same pathway. *Q*-value was calculated from a corrected *P*-value; the lower value means the greater intensiveness. Top 10 pathway terms enriched are shown. **(C)** Differential expression of genes clusters associated with the biosynthesis of secondary metabolites between ΔSte12 and Z7. Abbreviations: pks, polyketide synthase; nrps, non-ribosomal peptide synthetase; t1, Type1; and t3, Type 3.

To understand the global regulatory functions of AaSte12 and to identify candidate genes regulated by AaSte12, RNA-seq analyses were performed to compare the whole genome expression profiles between ΔSte12 and wild type (Figure 5A). In total, 664 DEGs, including 167 up-regulated and 497 downregulated genes (Supplementary Table S1) were identified in ΔSte12 (Adjusted FDR < 0.01). RT-PCR result of 7 randomly selected DEGs was consistent with RNA-seq data, indicating that RNA-seq data were reliable (Supplementary Figure S7). KEGG pathway enrichment statistics and GO analysis revealed that the downregulated genes were mostly associated with metabolic processes, catalytic activities and membrane functions. Top eight enriched pathways included betalain biosynthesis, isoquinoline alkaloid biosynthesis, melanogenesis, tyrosine metabolism, glyoxylate and dicarboxylate metabolisms, starch and sucrose metabolism, amino sugar and nucleotide sugar metabolisms, and MAPK signaling pathways (Figure 5B and Supplementary Figure S3).

**FIGURE 6:**
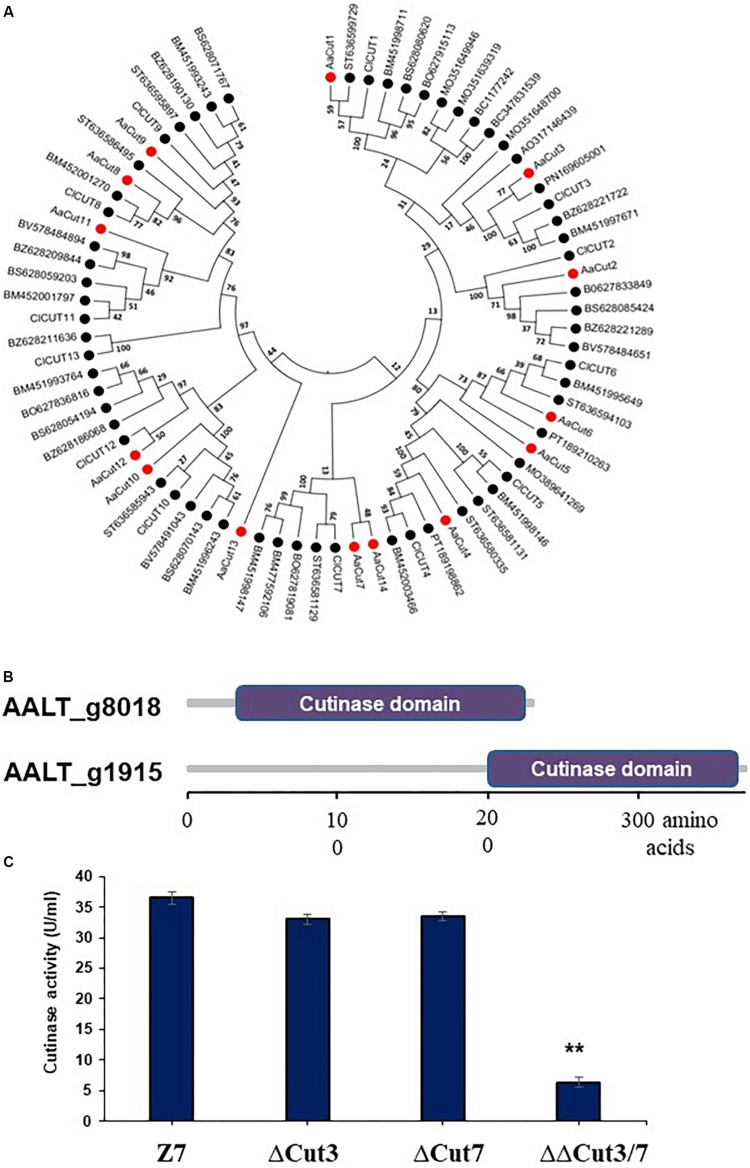
Characterization of cutinase-coding genes in *A. alternata*. **(A)** Unrooted phylogenetic tree showing the relationships of 14 cutinase proteins identified in the genome of *A. alternata* with other 62 orthogs identified from other plant pathogenic fungi. Amino acid sequences were aligned using Clustal W. Phylogenetic tree was constructed using the neighbor-joining method available in MEGA 6.0. **(B)** Schematic showing cutinase domains in two cutinases: Cut3 (AALT_g1915) and Cut7 (AALT_g8018), whose transcripts were decreased considerably in the AaSte12 deficiency strain. **(C)** Quantitative measurement of cutinase activities in the wild-type strain (Z7), the Cut3 deficiency strain (ΔCut3), the Cut7 deficiency (ΔCut7) strain, and the Cut3 Cut7 double mutation strain (ΔΔCut3/7). ^∗∗^Significant differences (*P* < 0.01).

Of 30 secondary metabolism gene clusters predicted by anti-SMASH, 11 were positively regulated by AaSte12 (Figure 5C), as all DEGs in these clusters were downregulated in ΔSte12 (Supplementary Table S2). Another six secondary metabolism gene clusters were negatively regulated by AaSte12. Of 283 transcription factors identified in the *A. alternata* genome, 23 were differentially expressed in ΔSte12; 22 were downregulated and only one was upregulated in ΔSte12 (Supplementary Table S3). Of 22 genes associated with conidiation in *A. alternata*, AALT_g9887 (LaeA in *A. nidulans*), AALT_g2799 (FlbA in *A. nidulans*) and AALT_g5513 (FlbC in *A. nidulans*) were significantly downregulated while AALT_g512 (VosA in *A. nidulans*) was upregulated in ΔSte12 (Supplementary Table S4).

**FIGURE 7:**
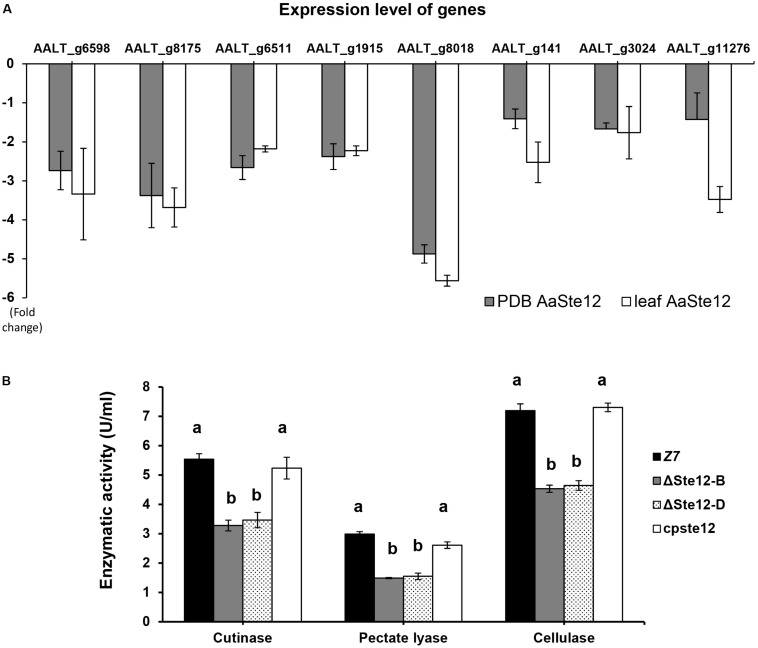
AaSte12 regulates the expression of genes encoding CWDEs and enzymatic activities. **(A)** RT-PCR analysis of genes encoding cutinases (AALT_g1915 and AALT_g8018), cellulases (AALT_g6511, AALT_g141), polygalacturonase (AALT_g3024), and pectate lyases (AALT_g6598, AALT_g8175 and AALT_g11276) identified from transcriptome analysis. The relative expression level of a gene in ΔSte12 was determined using a comparative C_T_ method in relation to that of wild type. Fungal strains were grown in PDB amended with or without citrus leaves. Hyphae were harvested for the isolation of RNA, which was used for cDNA synthesis and RT-PCR with gene-specific primers. **(B)** Quantification of cutinase, cellulase, and pectate lyases activities in wild type (Z7), two Ste12 deficiency strain (ΔSte12-B and D), and the cpSte12 strain expressing a functional copy of Ste12.

Of 675 genes encoding putative CAZymes identified in the *A. alternata* genome, 66 were differentially expressed in ΔSte12, of which nine genes were upregulated and 57 downregulated in ΔSte12 (Supplementary Table S5). Transcriptome analysis revealed that two genes encoding cutinases, three genes encoding pectate lyases, one gene coding for polygalacturonase, and two genes encoding cellulases were significantly downregulated in ΔSte12 (Supplementary Table S5). RT-PCR analysis of eight genes: two cutinase-coding genes (AALT_g1915 and AALT_g8018), three pectate lyase-coding genes (AALT_g6598, AALT_g8175, and AALT_g11276), two cellulase-coding genes (AALT_g6511 and AALT_g141), and one polygalacturonase-coding gene (AALT_g3024) revealed that the expression of these genes was all downregulated in ΔSte12 grown in PDB amended with or without citrus leaves (Figure 7A). In contrast, only a single gene (AALT_g11758) in the ACT biosynthetic gene cluster was upregulated (log_2_^FC^ = 1.37) and other ACT toxin biosynthetic genes were not differentially expressed in ΔSte12 (Supplementary Table S6).

### AaSte12 Is Required for the Production of Cell-Wall-Degrading Enzymes

The overall activities of cutinase, cellulase and pectate lyase produced by ΔSte12 were measured and compared to the observed activities of wild type. The results revealed a marked reduction of cutinase, cellulase and pectate lyase activities in two ΔSte12 strains (Figure 7B). Re-introducing and expressing a functional copy of *AaSte12* in ΔSte12 restored enzymatic activities to the wild-type levels.

### Cutinases Are Required for *A. alternata* Virulence in Citrus Leaves

**FIGURE 8:**
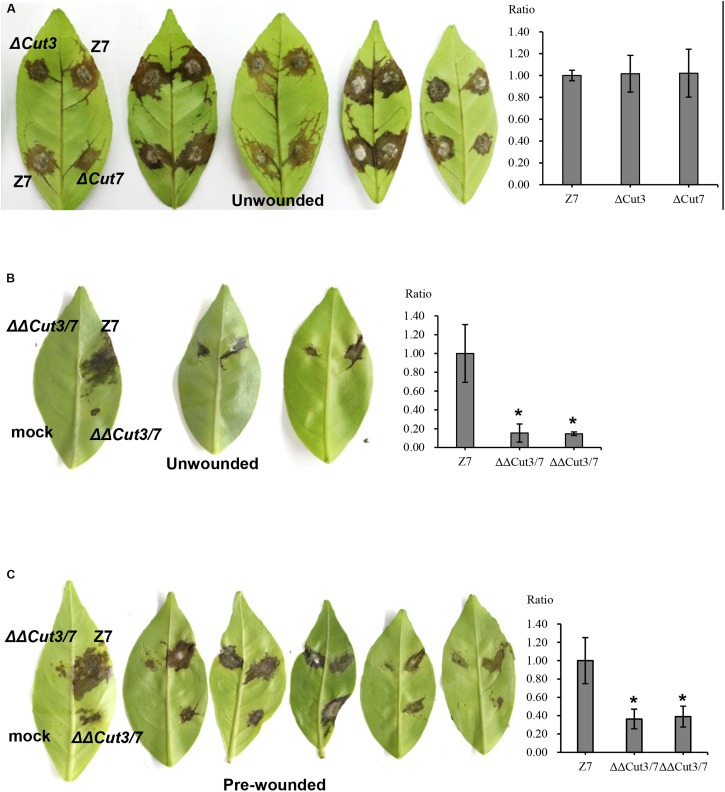
Cutinases are required for full virulence of *A. alternata* to infect citrus. Conidia were harvested from fungal strains: wild type (Z7), ΔCut3 (defective at Cut3), ΔCut7 (defective at Cut7), and ΔΔCut3/7 (Cut3 Cut7 double mutation) grown on PDA. Assays for fungal pathogenicity on detached Dancy leaves were performed by placing 10 μl conidial suspensions (10^4^ spores/ml) on each spot of the leaves. Spots treated with distill water were used as mock controls. **(A)** Dancy leaves (unwounded) inoculated with conidial suspensions of Z7, ΔCut3, or ΔCut7 all showed necrotic lesions 2 dpi. **(B)** Dancy leaves (unwounded) inoculated with the Cut3 Cut7 double mutation strain incited little or no visible lesion, while Z7 produced necrotic lesions. **(C)** Dancy leaves that were wounded before inoculation with the Cut3 Cut7 double mutation strain developed necrotic lesions, which were smaller than those induced by Z7. The percent changes of necrotic lesions appearing on the bottoms of the leaves calculated in relation to those induced by the wild-type strain Z7 are also indicated.

In total, 14 cutinase-, 18 pectate lyase- and 21 cellulase-coding genes were identified in the genome of *A. alternata* (Supplementary Figure S4 and Supplementary Table S7). SMART domain analysis confirmed the presence of a functional domain corresponding to each of the enzymes. Sequence alignment and maximum likelihood-based phylogenetic analyses revealed that the *A. alternata* genes encoding cutinases, cellulases, or pectate lyases were highly similar to those found in other fungi (Figure 6A and Supplementary Figure S4). Transcriptome analysis revealed that expression of two cutinase-coding genes *AaCut3* (AALT_g1915) and *AaCut7* (AALT_g8018) was drastically downregulated in ΔSte12. *AaCut3* was found to contain a 1110-bp intronless ORF encoding a 369 amino acid polypeptide. *AaCut7* was found to contain a 681-bp ORF interrupted by a small intron (53 bp) encoding a 226 amino acid polypeptide. A cutinase domain was found in both AaCut3 and AaCut7 (Figure 6B). A split marker approach was employed to determine the pathological roles of *AaCut3* and *AaCut7* (Supplementary Figures S5, S6). Mutational inactivation of either *AaCut3* or *AaCut7* resulted in fungal strains that displayed wild-type growth and conidiation, and produced wild-type levels of ACT (data not shown). ΔCut3 and ΔCut7 slightly but not significantly reduced cutinase activity compared to wild type (Figure 6C). However, the fungal strain (ΔΔCut3/7) carrying an *AaCut3* and *AaCut7* double mutation significantly reduced in cutinase activity. Pathogenicity assays on detached Dancy leaves (unwounded) revealed that ΔCut3 and ΔCut7 induced necrotic lesions at rates and magnitudes similar to those of wild type (Figure 8). In contrast, ΔΔCut3/7 displayed a marked reduction in virulence on Dancy leaves (unwounded) even though the mutant strain produced wild-type levels of ACT toxin (data not shown). When citrus leaves were wounded before inoculation, ΔΔCut3/7 induced necrotic lesions at sizes slightly larger than those on unwounded leaves. Compared to those induced by wild type, lesions induced by ΔΔCut3/7 on Dancy leaves with or without wounding were much smaller.

## Discussion

The Ste12 transcription regulator is a conserved protein commonly found in yeasts and filamentous fungi. Ste12 plays a role in mating and pseudohyphal growth in the budding yeast *S. cerevisiae* (Madhani and Fink, 1997). In filamentous fungi, Ste12-like proteins have been shown regulate fertility, development, and/or pathogenicity/virulence (Hoi and Dumas, 2010; Rispail and Di Pietro, 2010). In the current study, the results reveal that the *A. alternata Ste12* is required for the formation of conidia, the production of CWDEs, and fungal virulence on citrus. However, *AaSte12* is dispensable for vegetative growth, cellular resistance to osmotic and oxidative stress, and ACT toxin production. Importantly, the present study demonstrates the notion that not only is the HST produced by *A. alternata* required for fungal infection, but so too are CWDEs as key virulence determinants.

Transcriptome analysis of gene expression profiles between wild type and ΔSte12 reveals that the majority (>86%) of differentially expressed CAZymes-coding genes are positively regulated by AaSte12. Of them, two genes encoding cutinases, three genes encoding pectate lyases, one gene encoding polygalacturonase, and two genes encoding cellulases are positively regulated by AaSte12 based on transcriptome and RT-PCR analyses. Since no ACT toxin synthesis related genes were down-regulated in the transcriptome data, this leads to hypothesize that reduced virulence of ΔSte12 is likely a consequence of CWDE deficiency and that CWDEs play a role in virulence in the tangerine pathotype of *A. alternata*, which depends on the production of ACT toxin to kill host cells.

Although it has been well known that the ability to produce CWDEs is required for virulence of many plant pathogenic fungi (Kubicek et al., 2014), the role of CWDEs in virulence of toxin-producing fungi is not well understood. Many phytopathogenic fungi including *Alternaria* spp. can produce HSTs and the ability to produce HSTs has been demonstrated to be absolutely required for fungal pathogenesis (Walton, 1996; Tsuge et al., 2013). In addition, HSTs are important determinants of host range and specificity in particular plant species or cultivars. Thus, the pathological role of CWDEs in toxin-producing fungi of plants is often masked by the important role of HST in fungal pathogenesis. In the present study, the *A. alternata Ste12* deficient strain displays a reduced virulence and CWDEs but retains the ability to produce ACT toxin, implicating the involvement of CWDEs in *A. alternata* pathogenesis to citrus. This assumption is confirmed further by genetic analysis of cutinase deficient mutants.

Citrus cuticle consisting of wax, cellulose, and cutin (Baker et al., 1975) is the first barrier to fungal invasion. Thus, the ability to breach the cuticle of citrus may play a role in virulence of *A. alternata*. Deletion of either *AaCut3* or *AaCut7* has no drastic effects on cutinase activity and fungal virulence. However, a concomitant deletion of both *AaCut3* and *AaCut7* results in a fungal strain (ΔΔCut3/7) that is severely defective for the production of cutinases and the induction of necrotic lesions on citrus leaves. The results clearly demonstrate that cutinases play an important role in *A. alternata* pathogenesis to citrus. ΔΔCut3/7 apparently induces less severe lesions on Dancy leaves even the leaves are wounded before inoculation. Thus, cutinases are required not only for breakdown of leaf cuticle layers during penetration but also for degradation of plant cell wall during colonization and hyphal invasion. Cutinases have been suggested to be required for carbon acquisition during saprophytic growth of fungi (Koller and Parker, 1989). Moreover, cutin monomers released by cutinases may trigger virulence-associated signaling pathways within *A. alternata* as proposed in other fungal pathogens (Woloshuk and Kolattukudy, 1986; Wang et al., 2000).

The role of cutinases in virulence varies, sometimes controversial, among phytopathogenic fungi, highly depending on fungal life styles and their interactions with host plants. Cutinases have been shown to be required for virulence in a number of fungal pathogens including: *Fusarium solani* f. sp. *pisi* (Rogers et al., 1994), *Pyrenopeziza brassicae* (Li et al., 2003), *Colletotrichum gloeosporioides* (Wang et al., 2017), *C. truncatum* (Auyong et al., 2015), *Monilinia fructicola* (Lee et al., 2010), *Curvularia lunata* (Liu et al., 2016), and *Rhizoctonia cerealis* (Lu et al., 2018). In the rice blast fungus *M. grisea*, cutinase 2 but not cutinase 1 is required for appressorium differentiation and host penetration (Skamnioti and Gurr, 2007). In contrast, cutinases apparently play no role in virulence of *F. solani* f. sp. *cucurbitae* (Crowhurst et al., 1997) and *Botrytis cinerea* (Van Kan et al., 1997). The tangerine pathotype of *A. alternata* does not form specialized infection structures to breach the host cuticle. Secretion of cutinases or other CWDEs by *A. alternata* may facilitates the hydrolysis of citrus cuticle, enabling the ACT and pathogen to enter host tissue. In addition to cutinases, deletion of *AaSte12* also impacts cellulase and pectate lyase activities. It is very likely that those enzymes may also have a role in *A. alternata* virulence, even though an endopolygalacturonase (endoPG)-coding gene plays no role in the tangerine pathotype of *A. alternata* (Isshiki et al., 2001). This is likely due to the fact that a given CWDE is often encoded by multiple genes in fungi. Since ΔΔACTT6 failed to induce necrotic lesions on wounded or unwounded citrus leaves (data not shown), ACT toxin is still more important than Ste12 or CWDEs in terms of virulence of *A. alternata*.

Ste12 proteins have been shown to be required for mating and the formation of sexual structures in yeasts and filamentous fungi, including *S. cerevisiae* (Madhani and Fink, 1997), *Candida albicans* (Liu et al., 1994), *Cryptococcus neoformans* (Yue et al., 1999), *Cryphonectria parasitica* (Deng et al., 2007), *B. cinerea* (Schamber et al., 2010), *Neurospora crassa* (Li et al., 2005), *Sordaria macrospora* (Nolting and Pöggeler, 2006), and *A. nidulans* (Vallim et al., 2000). Several genes whose products are associated with spore formation in fungi are differentially expressed in ΔSte12, which is completely devoid of conidiation. The tangerine pathotype of *A. alternata* does not have a known sexual cycle. Conidia are essential for dispersal, for the initiation of surface penetration on citrus, and the completion of life cycle. The formation of conidia in the tangerine pathotype of *A. alternata* is a complex process, which is modulated by multiple signaling pathways, including cAMP-dependent protein kinase A-, the NADPH oxidase complex (Nox)-, the thioredoxin and glutaredoxin-, the Fus3 and Slt2 MAPKs-, as well as the calcineurin phosphatase and phospholipase C-mediated signaling pathways (Yang and Chung, 2012; Tsai et al., 2013; Chung, 2014; Tsai and Chung, 2014; Yang et al., 2016; Ma et al., 2018). However, in other filamentous fungi including *F. graminearum* (Gu et al., 2015), *Metarhizium acridum* (Wei et al., 2017) and *M. grisea* (Park et al., 2002), Ste12 mutants are not affected in conidial formation. In contrast, *Ste12* homolgs of *A. brassicicola* (Cho et al., 2009), *N. crassa* (Li et al., 2005), *B. cinerea* (Schamber et al., 2010) and *Mycosphaerella graminicola* (Kramer et al., 2009) have been reported to be associated with conidial development. Thus, the conserved *Ste12* could have divergent roles in terms of conidial formation in different fungi.

Since Ste12 is presumably an immediate target downstream of Fus3 MAPK, it is not surprising that deletion of Ste12 leads to a complete attenuation of conidial formation. However, in addition to conidiation, the *A. alternata* Fus3 kinase has previously been characterized to be required for growth, pathogenicity, the production of melanin and hydrolytic enzymes, as well as resistance to copper fungicides and osmotic stress (Lin et al., 2010). In addition, Fus3 has been reported as a negative regulator of cutinase (Lin et al., 2010), which means the regulatory mechanisms of Fus3 and Ste12 on cutinase activities may be different. Because phenotypes resulted from Fus3 or Ste12 deletion are not completely identical, it appears that Ste12 regulates only a subset of the Fus3-dependent functions. Even though, transcriptome analysis suggests that AaSte12 plays a diverse role in a wide range of biological functions in *A. alternata*. Those may include metabolisms associated with tyrosine, starch, sucrose, amino sugar, and nucleotide sugar, as well as glyoxylate and dicarboxylate. AaSte12 may also be required for the biosynthesis of melanin, isoquinoline alkaloid, and betalain, all of which warrant further research.

In summary, this study provides genetic evidence to define the biological and pathological functions of AaSte12 and cutinases in the tangerine pathotype of *A. alternata*, which produces a host selective toxin to kill host cells and acquires nutrients exclusively from dead tissue. AaSte12 is required for conidiation, production and secretion of CWDEs (cutinases, cellulases, and pectate lyases), and fungal virulence. Reduced virulence in two AaSte12-deficient mutants is attributable to CWDE rather than toxin deficiency. This study expands our understanding of how *A. alternata* conquers plant obstacles to achieve successful penetration and colonization using multiple arsenals. Overall, this study further underlines an important role of CWDEs in fungal pathogenesis even for a toxin-producing pathogen.

## Data Availability Statement

The datasets generated for this study can be found in the SRR8556263, SRR8556264, SRR8556261, and SRR8556262.

## Author Contributions

HM, HL, and K-RC designed the experiments and wrote the manuscript. HM analyzed the data, obtained all the mutants in this manuscript, and performed the experiments of ΔSte12. BZ performed the experiments of cutinase mutants and HPLC. YG and XS analyzed the data. All authors reviewed the manuscript.

## Conflict of Interest

The authors declare that the research was conducted in the absence of any commercial or financial relationships that could be construed as a potential conflict of interest.

## Footnotes

1 http://bcb.unl.edu/dbCAN2/blast.php
