# Supplementary material for: Cell-Wall-Degrading Enzymes Required for Virulence in the Host Selective Toxin-Producing Necrotroph Alternaria alternata of Citrus
Source: Front Microbiol. 2019 Nov 22;10:2514. doi: 10.3389/fmicb.2019.02514 (PMC6883767; doi:10.3389/fmicb.2019.02514)
Supplement: Supplementary file 2 [file Presentation_1.pptx]

## Slide 1
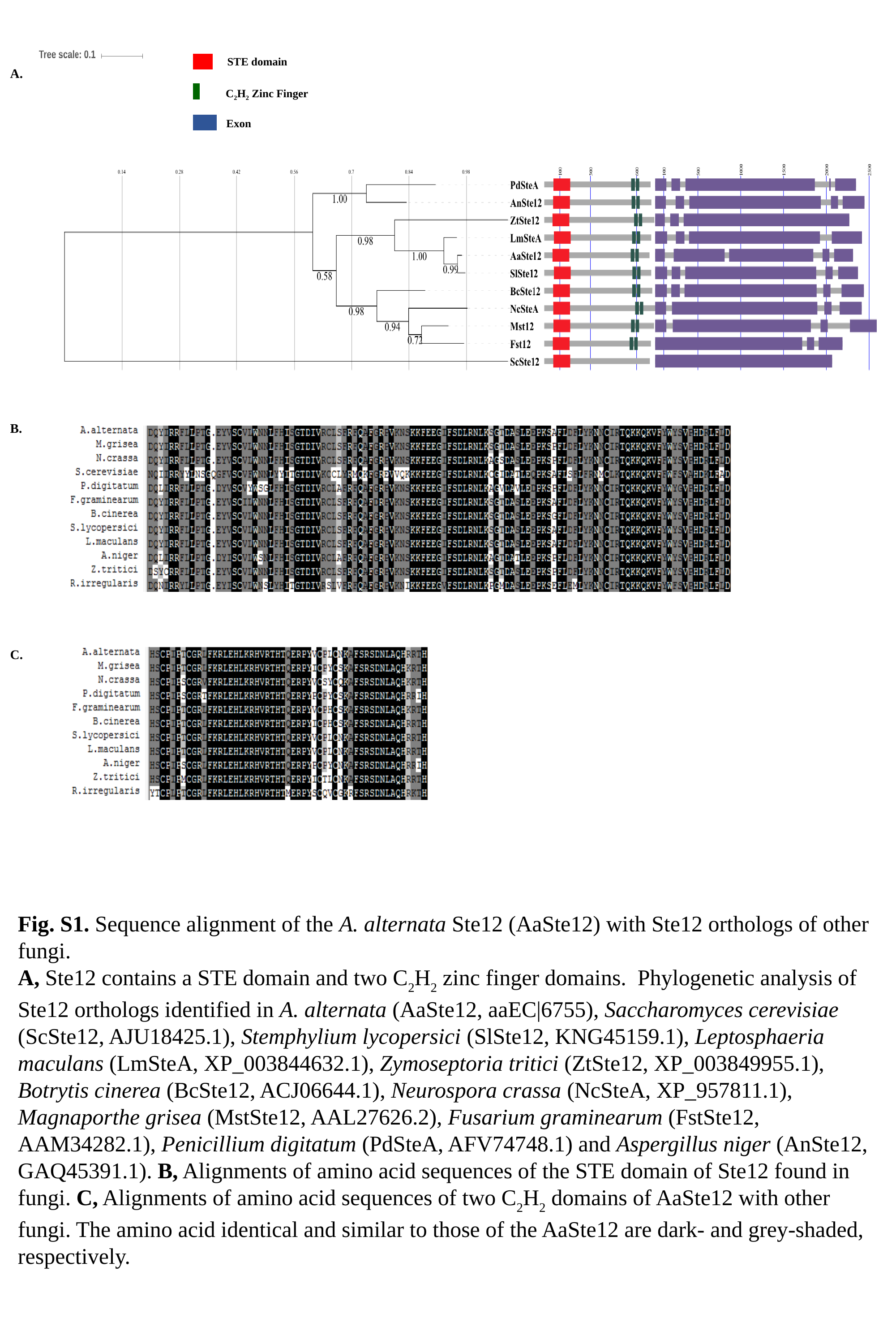

STE domain
A.
C2H2 Zinc Finger
Exon
B.
C.
Fig. S1. Sequence alignment of the A. alternata Ste12 (AaSte12) with Ste12 orthologs of other fungi.
A, Ste12 contains a STE domain and two C2H2 zinc finger domains. Phylogenetic analysis of Ste12 orthologs identified in A. alternata (AaSte12, aaEC|6755), Saccharomyces cerevisiae (ScSte12, AJU18425.1), Stemphylium lycopersici (SlSte12, KNG45159.1), Leptosphaeria maculans (LmSteA, XP_003844632.1), Zymoseptoria tritici (ZtSte12, XP_003849955.1), Botrytis cinerea (BcSte12, ACJ06644.1), Neurospora crassa (NcSteA, XP_957811.1), Magnaporthe grisea (MstSte12, AAL27626.2), Fusarium graminearum (FstSte12, AAM34282.1), Penicillium digitatum (PdSteA, AFV74748.1) and Aspergillus niger (AnSte12, GAQ45391.1). B, Alignments of amino acid sequences of the STE domain of Ste12 found in fungi. C, Alignments of amino acid sequences of two C2H2 domains of AaSte12 with other fungi. The amino acid identical and similar to those of the AaSte12 are dark- and grey-shaded, respectively.

## Slide 2
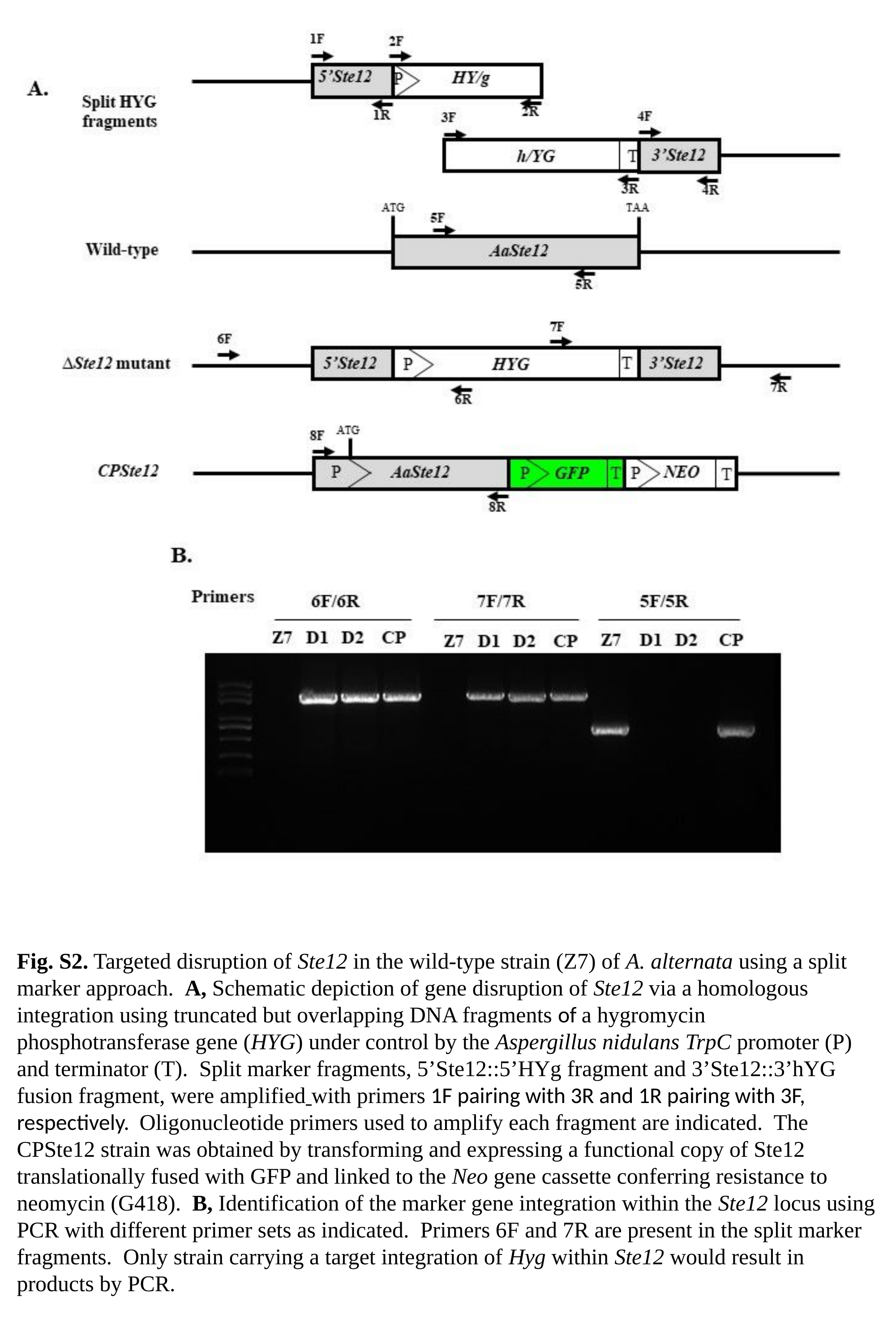

Fig. S2. Targeted disruption of Ste12 in the wild-type strain (Z7) of A. alternata using a split marker approach. A, Schematic depiction of gene disruption of Ste12 via a homologous integration using truncated but overlapping DNA fragments of a hygromycin phosphotransferase gene (HYG) under control by the Aspergillus nidulans TrpC promoter (P) and terminator (T). Split marker fragments, 5’Ste12::5’HYg fragment and 3’Ste12::3’hYG fusion fragment, were amplified with primers 1F pairing with 3R and 1R pairing with 3F, respectively. Oligonucleotide primers used to amplify each fragment are indicated. The CPSte12 strain was obtained by transforming and expressing a functional copy of Ste12 translationally fused with GFP and linked to the Neo gene cassette conferring resistance to neomycin (G418). B, Identification of the marker gene integration within the Ste12 locus using PCR with different primer sets as indicated. Primers 6F and 7R are present in the split marker fragments. Only strain carrying a target integration of Hyg within Ste12 would result in products by PCR.

## Slide 3
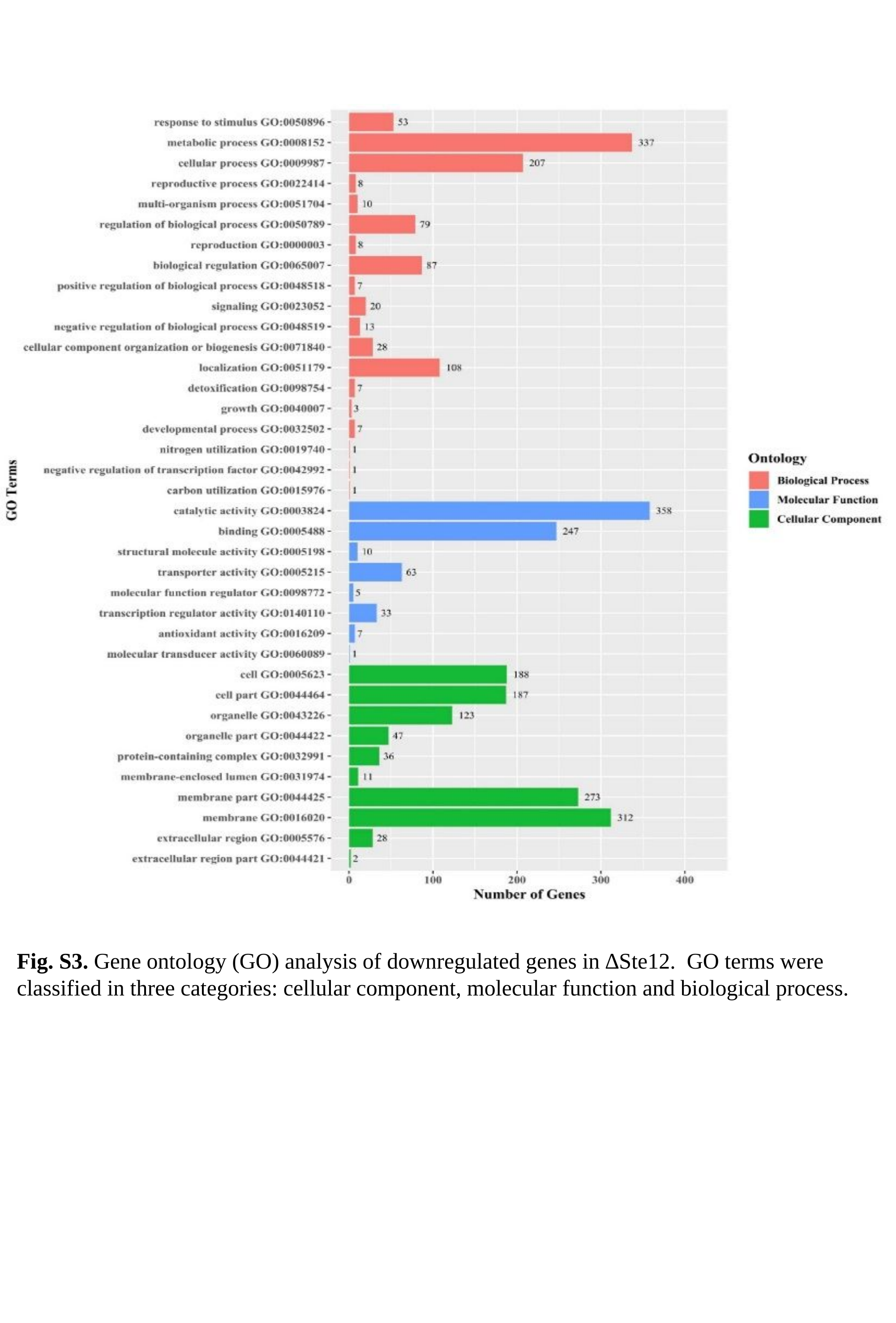

Fig. S3. Gene ontology (GO) analysis of downregulated genes in ∆Ste12. GO terms were classified in three categories: cellular component, molecular function and biological process.

## Slide 4
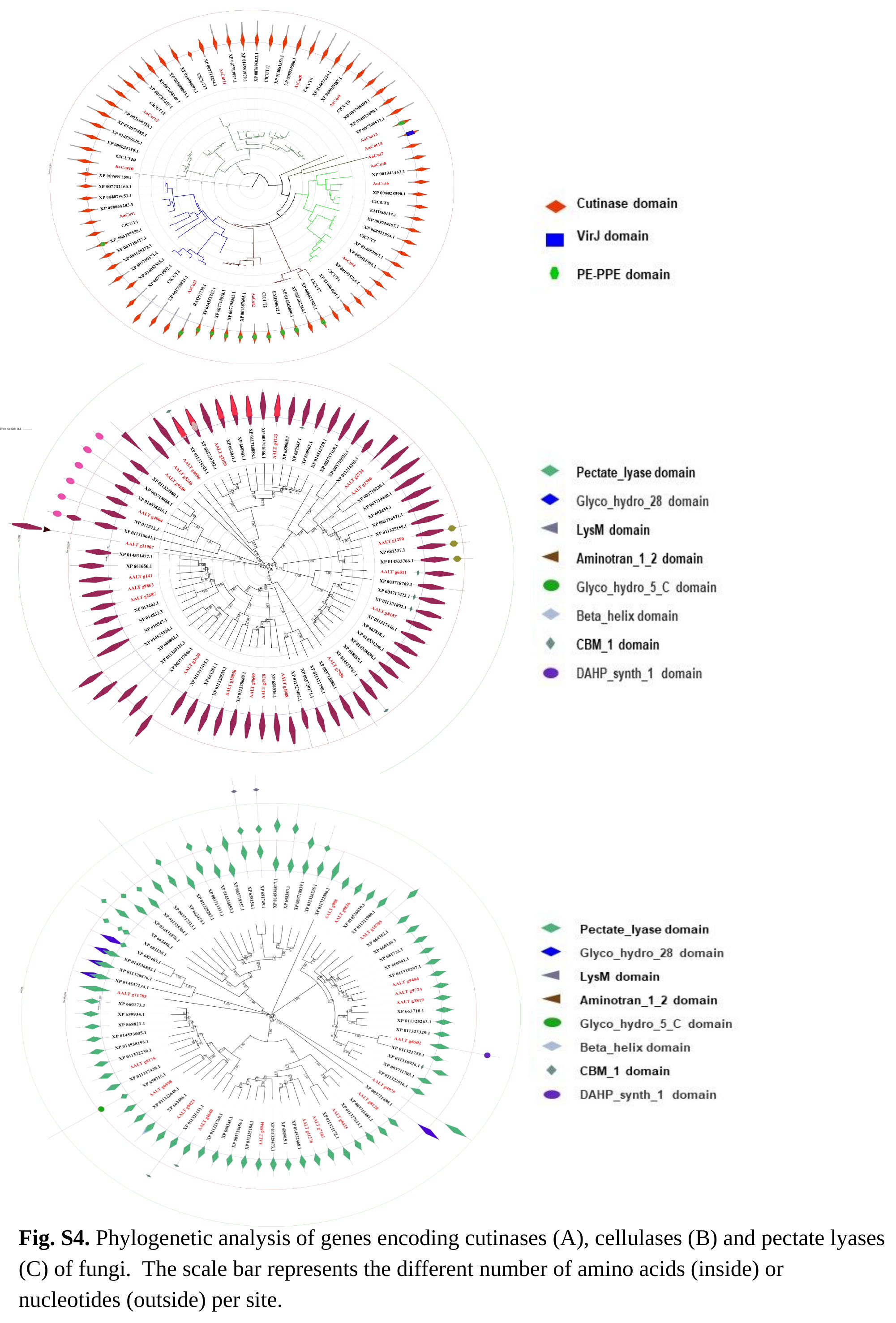

Fig. S4. Phylogenetic analysis of genes encoding cutinases (A), cellulases (B) and pectate lyases (C) of fungi. The scale bar represents the different number of amino acids (inside) or nucleotides (outside) per site.

## Slide 5
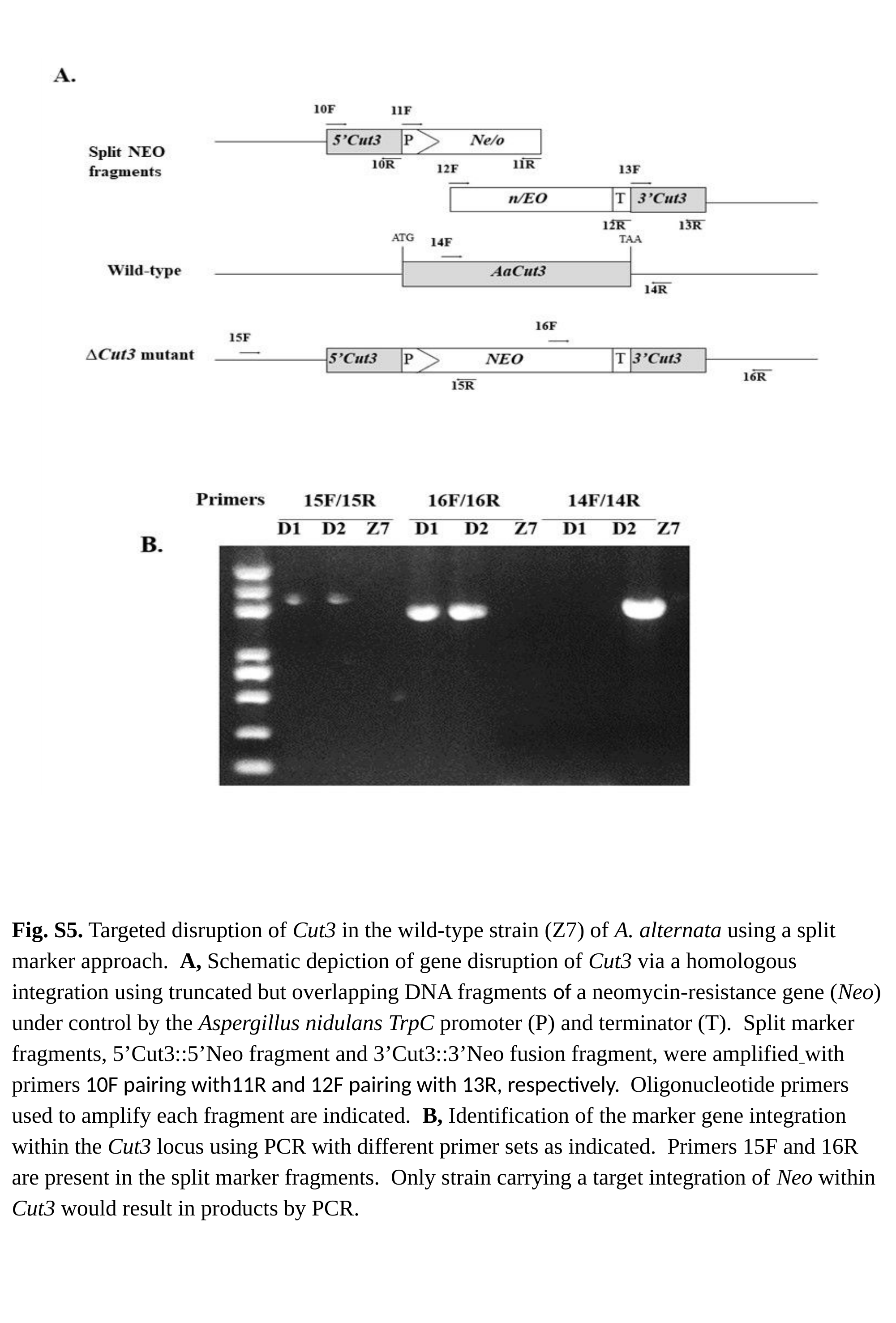

Fig. S5. Targeted disruption of Cut3 in the wild-type strain (Z7) of A. alternata using a split marker approach. A, Schematic depiction of gene disruption of Cut3 via a homologous integration using truncated but overlapping DNA fragments of a neomycin-resistance gene (Neo) under control by the Aspergillus nidulans TrpC promoter (P) and terminator (T). Split marker fragments, 5’Cut3::5’Neo fragment and 3’Cut3::3’Neo fusion fragment, were amplified with primers 10F pairing with11R and 12F pairing with 13R, respectively. Oligonucleotide primers used to amplify each fragment are indicated. B, Identification of the marker gene integration within the Cut3 locus using PCR with different primer sets as indicated. Primers 15F and 16R are present in the split marker fragments. Only strain carrying a target integration of Neo within Cut3 would result in products by PCR.

## Slide 6
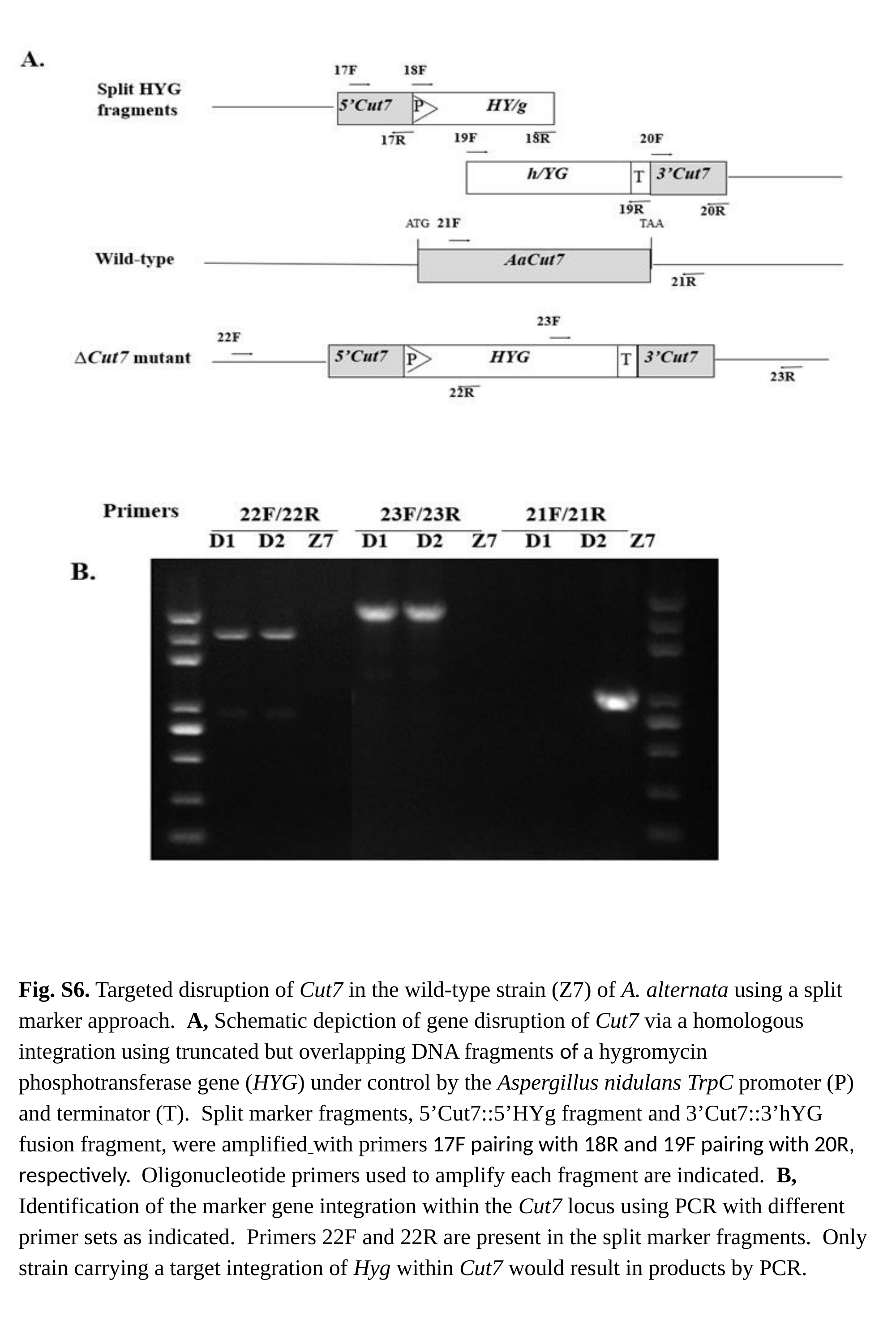

Fig. S6. Targeted disruption of Cut7 in the wild-type strain (Z7) of A. alternata using a split marker approach. A, Schematic depiction of gene disruption of Cut7 via a homologous integration using truncated but overlapping DNA fragments of a hygromycin phosphotransferase gene (HYG) under control by the Aspergillus nidulans TrpC promoter (P) and terminator (T). Split marker fragments, 5’Cut7::5’HYg fragment and 3’Cut7::3’hYG fusion fragment, were amplified with primers 17F pairing with 18R and 19F pairing with 20R, respectively. Oligonucleotide primers used to amplify each fragment are indicated. B, Identification of the marker gene integration within the Cut7 locus using PCR with different primer sets as indicated. Primers 22F and 22R are present in the split marker fragments. Only strain carrying a target integration of Hyg within Cut7 would result in products by PCR.

## Slide 7
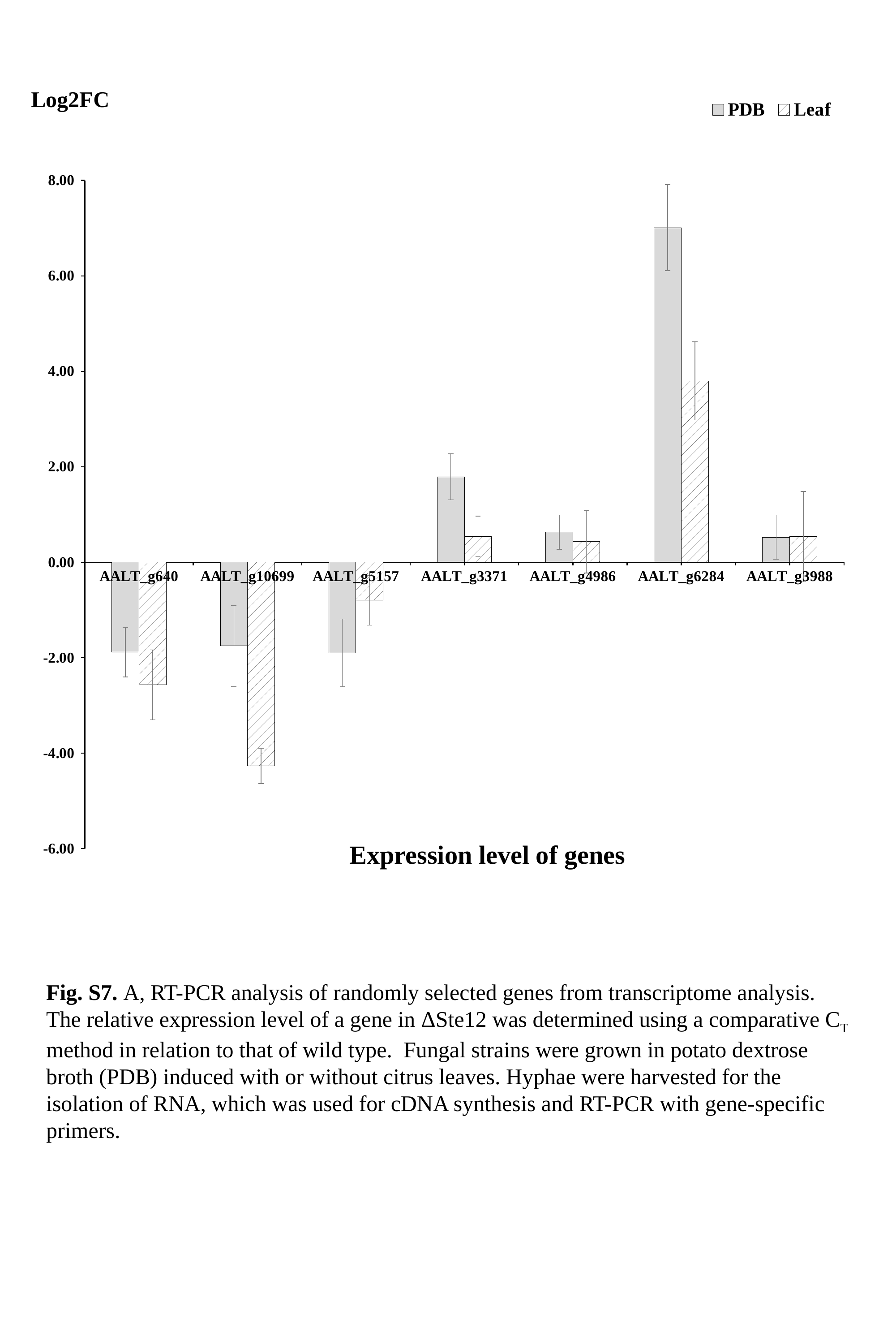

### Chart: Log2FC
| Category | PDB | Leaf |
|---|---|---|
| AALT_g640 | -1.8837396302678993 | -2.5687882165387337 |
| AALT_g10699 | -1.754493568424999 | -4.265588668868355 |
| AALT_g5157 | -1.9001101456911655 | -0.7973517410604009 |
| AALT_g3371 | 1.791111136269001 | 0.5429261999326123 |
| AALT_g4986 | 0.6324390694441296 | 0.4332417092909253 |
| AALT_g6284 | 7.010027957651034 | 3.7986359691247245 |
| AALT_g3988 | 0.5241413074266342 | 0.5369104795782652 |Expression level of genes
Fig. S7. A, RT-PCR analysis of randomly selected genes from transcriptome analysis. The relative expression level of a gene in ΔSte12 was determined using a comparative CT method in relation to that of wild type. Fungal strains were grown in potato dextrose broth (PDB) induced with or without citrus leaves. Hyphae were harvested for the isolation of RNA, which was used for cDNA synthesis and RT-PCR with gene-specific primers.
